# Social Determinants of Health in Older Adults

**DOI:** 10.1093/geroni/igaf122.1653

**Published:** 2025-12-31

## Abstract

Social determinants of health impact a variety of health outcomes in older adults. and often start early in life. This session provides insight into the impact of the environment, nutrition and support systems can affect health in older adults.

